# Irish and Turkish pre-service teachers understanding and perceptions of enterprise education

**DOI:** 10.1016/j.heliyon.2021.e07591

**Published:** 2021-07-19

**Authors:** Peter Tiernan, İsa Deveci

**Affiliations:** a School of STEM Education, Innovation and Global Studies, Institute of Education, Dublin City University, Dublin, Ireland; b Faculty of Education, Kahramanmaras Sutcu Imam University, Kahramanmaraş, Turkey

**Keywords:** Enterprise education, Entrepreneurship education, Teacher education

## Abstract

While many of its roots are derived from business contexts, enterprise education is evolving, and interest in its impact on initial teacher education is increasing. While common goals exist across regions, practices tend to differ. The perceptions of pre-service teachers who have been exposed to enterprise education in different countries can provide important clues about developing entrepreneurial teachers through their initial teacher education. This paper examines the perceptions of Irish and Turkish pre-service teachers who are exposed to enterprise education. The research data were obtained through semi-structured interviews with three main categories and 14 open-ended questions. The data collected from interviews was analysed using the constant comparative method. Findings show that enterprise education has a positive impact on pre-service teachers regarding content knowledge, pedagogical knowledge and future intentions. Findings also indicate that exposure to enterprise education may affect pre-service teachers' perceptions of its role in education.

## 1 Introduction

The development of an enterprising mindset has been advocated by high-level European policy documentation for many years (for example: European Commission, 2000; European Commission, 2003; European Commission, 2011). Employers and professional bodies also advocate for the development of enterprising skills and behaviours in university graduates (Finch et al., 2013), recognising that an entrepreneurial mindset can improve organisational adaptivity (Täks, 2015), creativity and problem solving (Birdthistle et al., 2016), risk taking (Edwards et al., 2014), and initiative (Bell, 2016). Government policy in Ireland and Turkey state similar positions: The Government of Ireland (2014) have ambitions for Ireland “to be among the most entrepreneurial nations in the world”; The Turkish Government aimed at developing entrepreneurship education policies to enable young individuals to be entrepreneurs (Ministry of National Education, 2018; Council of Higher Education, 2018). It is widely accepted that education plays a significant role in the development of enterprising attitudes and behaviours (European Commission, 2004, 2008) and as such, the education system in both jurisdictions have been identified as a critical player in the development of a more enterprising society. Ireland's Further Education and Training Authority's (SOLAS) action plan (2014) committed to identifying best practice education and training for entrepreneurship within Further Education and Training provision. Similarly, in Turkey, the entrepreneurship action plan 2015–2018 sought to promote an entrepreneurship culture by increasing the knowledge and skills of teachers with regard to entrepreneurship. There have been repeated calls for the integration and investigation of enterprise education across all levels of the education system (European Commission, 2004, 2008, 2012a, 2012b). Higher Education Institutions have traditionally been active in the area, with many universities embracing an entrepreneurial vision and incorporating add-on entrepreneurship programmes and initiatives (NIRAS, 2008; Rae et al., 2010). However, recent Organisation for Economic Co-operation and Development (OECD) reviews of institutional support of entrepreneurship across member states recommend increased participation and engagement from students across the subject spectrum, arguing that enterprise education should be expanded across all subject disciples and move away from a solely business focus (OECD, 2017a; OECD, 2017b; OECD, 2017c; OECD, 2018; OECD, 2019a; OECD, 2019b). Relevant literature examining the views of pre-service teachers remains particularly low (Arruti and Panos-Castro, 2020). Studies by Tiernan (2016) and Deveci (2016) have looked at pre-service teachers' attitudes and perceptions of enterprise and entrepreneurship, findings indicated that while pre-service teachers did display an understanding of these concepts, further work was needed to develop their knowledge of enterprise education and how this applies to their role as educators.

Additionally, Arruti and Panos-Castro (2020) investigated whether participating in an entrepreneurship education program with short-term international placements would help pre-service teachers in primary education degrees. Our research will contribute to the literature in the area by determining the perceptions of pre-service teachers from different cultural backgrounds, to enterprise education. Specifically, the research will reveal the perceptions of pre-service teachers from international education faculties in İreland and Turkey. In addition, the findings of this study may contribute to the efforts of other countries to train entrepreneurial teachers.

## 2 Theoretical framework

Enterprising behaviours can bring about societal changes (European Commission, 2003) and impact positively on individuals’ career and personal aspirations (Matlay, 2008). Participation in enterprise education has the potential to develop a wide range of transferable attributes in the cognitive, behavioural and affective domains. These include, but are not limited to: the ability to recognise and exploit opportunities (Rae, 2007; Burduş, 2010); the ambition and drive to start something new (Burduş, 2010); tolerance for ambiguity and uncertainty (Kirby, 2007); creativity and innovation (Seelig, 2012); calculated risk-taking (Caird, 1991; Burduş, 2010); the confidence to propose ideas (Gibb, 2007) and take initiative (Hegarty and Jones, 2008); the ability to manage themselves and manage others (Burduş, 2010); and the flexibility to adapt to change (Burduş, 2010).

In order to fully understand the concept of enterprise education, it is necessary to examine the wider context within which it sits. Enterprise education and entrepreneurship education are often used interchangeably, however they can also hold different meanings to different scholars and in different jurisdictions. For example, entrepreneurship education is sometimes defined in terms of promoting awareness and orientation towards business creation (Kirby, 2004), with the purpose of equipping participants with the knowledge, attributes and capabilities required to set up a new venture or business (Jones et al., 2014; Jones and Iredale, 2010). Some scholars refer to this form of education as ‘narrow-focus’ with the objective of ‘becoming an entrepreneur’ (Fayolle and Gailly, 2012). Enterprise education on the other hand is generally defined in terms of promoting the development of a set of transferable skills, attitudes and behaviours which can be applied in a wide range of life contexts (Bridge et al.*, 2010*), which is sometimes referred to as ‘broad-focus’ entrepreneurship education (Mwasalwiba, 2010). The overlapping nature of enterprise education and entrepreneurship education can lead to misunderstandings and a lack of clarity on its objectives and focus (Hannon, 2005; Pittaway and Cope, 2007). For instance, Kirby (2004) suggests that while there is no universally accepted definition of an entrepreneur, simply equating one with new venture creation is too narrow, instead proposing they be viewed as individuals who see and act upon opportunity in a variety of contexts. Others identify entrepreneurs as individuals with enterprising skills and behaviours that enable them to act in innovative ways (for example, Hameed and Irfan, 2019; Rae, 2006). Gibb (1993) therefore contends that enterprise education and entrepreneurship education are conceptually the same but contextually different. Thus, enterprise education has been taken into account since the current research is conducted within the educational context.

The concept of enterprise education has become embedded in many educational institutions, and the myth that entrepreneurs are ‘born’ that way, has largely been dispelled (Leffler and Näsström, 2014; Hindle, 2007). However, research indicates that there is much variation in the approaches used in the teaching of enterprise education. Lectures and other traditional approaches are used to imbue the theoretical and cognitive aspects (Hynes, 1996), while experiential activities such as problem solving and project-based learning are adopted for developing enterprising skills and behaviours (Ruskovaara and Pihkala, 2013). Additionally, strategies such as case studies and guest speakers are incorporated to establish links between material being studied and the real world, helping to provide context upon which students can draw (Solomon, 2007). Reviewing literature on entrepreneurship education, Mwasalwiba (2010) found that the most common approaches are lectures, case studies and group discussion, with experiential methods being far less common. According to Hindle (2007), more attention should be given to developing enterprising skills and behaviours through imaginative approaches which promote both analytical and lateral thinking (Kirby, 2004). Similarly, Lackéus (2015) argues that enterprise education programmes that are based on active engagement and contain a practical focus, are more successful than purely theoretical interventions. Furthermore, rather than consisting of generic content, research indicates that enterprise education should be contextually relevant to the students and take into consideration the students' field of study and their future career aspirations (Hynes, 1996; Jones and Iredale, 2010).

The role of the teacher is crucial in enterprise education as it is their understanding of the concepts and agency in the process that facilitates the development of enterprising skills in their students (McAuliffe and Winter, 2013). Research indicates that participation in enterprise education at the initial teacher education stage increases awareness and receptiveness to entrepreneurship (Anagün and Atalay, 2017) and helps them to internalise it and incorporate it into their future practice (Lepistö and Ronkko, 2013). However, Deveci (2017) cautions that without adequate exposure to elements such as risk-taking, leadership and ambiguity, teachers do not fully understand the nature of entrepreneurship. Comparative studies are a useful tool in identifying similarities and differences in experiences and perceptions from disparate jurisdictions. Examples include studies by Davies et al. (2004) which investigated teachers’ perceptions of citizenship and enterprise in England and Hungary; and Davies and Issitt (2005) which examined citizenship education in Australia, Canada and England. Our research draws on this comparative approach to reveal the similarities and differences in the perceptions of Turkish and Irish pre-service teachers who are exposed to enterprise education. For the purpose of this research, the sub-problems addressed are as follows:

• What are pre-service teachers' perceptions of content knowledge of enterprise education?
• What are pre-service teachers' perception of pedagogical knowledge and approaches to enterprise education?
• What are pre-service teachers' perception of activation and likelihood of using enterprise education skills in their future.

## 3 Methodology

The purpose of this study was to gather the perceptions and opinions of participating pre-service teachers and as such, a qualitative research approach was adopted. Phenomenology and phenomenography are frequently used by educational researchers who want to understand the lived experiences of students, especially in certain educational contexts (Stolz, 2020). In phenomenology, researchers focus on the essence of the experience, while in phenomenography the perception regarding the relevant phenomenon is examined, and experience may not be used as the starting point of the research (Marton, 2005; Marton and Pang, 2008). In addition, phenomenographic research is used to examine how individuals' experiences affect and change their reactions to phenomena (Marton and Pang, 2008). Within the scope of this research, the phenomenological research method was not preferred because the researchers did not directly focus on the perceptions of the participants based on their real business experiences. Thus, a phenomenographic method was used to determine what kind of similarities and differences enterprise education creates in the perceptions of pre-service teachers.

### 3.1 Sample

This study was carried out in the Institute of Education at Dublin City University, Ireland and in the Faculty of Education at Kahramanmaraş Sütçü İmam University, Turkey. Research data in both countries were collected in the spring semester 2017–2018. Ethical approval was granted by Kahramanmaraş Sütçü İmam University ethics committee and informed consent was obtained from all participants. Eight Irish participants (male = 4, female = 4) were drawn from the ‘Further Education’ strand of the B.Sc. in Education and Training, which is an optional strand, enabling pre-service teachers to work in Further Education upon graduation, allowing them to teach a range of subjects in the Further Education sector in Ireland. Participants were completing the module ‘enterprise education and team learning’ during semester two of final year. This was a compulsory module, taught by author 1, two hours per week. Additionally, 11 Turkish pre-service teachers (male = 4, female = 7) participated in this research. Turkish participants were drawn from B.Sc. in science teacher education department which allows pre-service teachers to become science teachers. The pre-service teachers were completing the module called “entrepreneurship in science education”, taught by author 2, in the second semester of the final year. Both participant groups were drawn from education faculties and were training for roles as educators in their respective countries. Enterprise education is not featured in the compulsory education system in Ireland or Turkey, thus participants had no prior experience of or engagement with enterprise education before university or as part of their previous studies at the authors' universities. All participants were volunteer interviewees from the modules which they were attending. Thus, convenience sampling was used as a selection mechanism, as it provided researchers with ease of access to the participants (Miles et al., 2014). The sample size was considered sufficient for the qualitative study, since the desired sample size for phenomenographic studies is relatively small (Jobin and Turale, 2019).

### 3.2 The modules

Both modules were designed to give pre-service teachers an understanding of the theory and practice of enterprise education, leadership, and the role of teams. Pre-service teachers were encouraged to develop their own skills, attitudes and behaviours, and consider these approaches for use as teachers in the future. Module topics were ‘what is enterprise education’, ‘enterprise education policy and practice’, ‘effective teams in enterprise education’, ‘leadership’, ‘decision making and challenges’ and ‘entrepreneurial project’.

### 3.3 Description of process

The purpose of the modules was to increase pre-service teachers' understanding of, and experience with enterprise education, leadership, and the role of teams.

In the Irish grouping, content for lectures was divided into a number of sections using a range of strategies containing: Theory – tackling the historical, contextual and contemporary perspectives; Case studies – providing examples of approaches, behaviours and skills in a variety of real world contexts, focussing particularly on education; Discussion – providing opportunities for pre-service teachers to link their understanding of enterprise education, leadership and teams to case studies and theoretical information previously covered. Group projects – pre-service teachers collaborated in small groups to identify problems or opportunities in education. Groups then worked together to formulate a solution to the problem or plan for realising the opportunity identified; Finally, short reflective activities took place at the end of each lecture to consolidate learning by examining the links between theory, case studies and practical work.

In the Turkish grouping, practice and theory were carried out together. Theory: Concepts such as entrepreneur, entrepreneurship, entrepreneurial training, entrepreneurship skills, science education and entrepreneurship are explained, how entrepreneurship can be also integrated with science subjects was explained. Practice: Entrepreneurial project – life stories of entrepreneurial individuals, introduced sample entrepreneurial projects, the focus was on what kind of entrepreneurial projects could be developed from science classrooms, Discussion – innovative project ideas discussed, pre-service teachers were allowed to produce innovative and entrepreneurial projects (participants were asked to find problems that would create value by thinking about the problems they observed in their daily lives.), Group activities – pre-service teachers were allowed to work in teams during entrepreneurial project (the participants found a problem in groups of 3–5 participants, found different solutions to the problem, decided on one of the solutions), Finally, pre-service teachers introduced their project ideas.

### 3.4 Instruments

Data collection was carried out using semi-structured interviews. Data gathered was qualitative in nature. Questions were asked with the following categories in mind: 1) Content knowledge of enterprise education; 2) Pedagogical knowledge of enterprise education; 3) Activation and likelihood of using enterprise education skills in the future. In the first category, content knowledge, pre-service teachers were asked open-ended questions to determine their perceptions about both the enterprise education and characteristics of an enterprising person. In the second category, pedagogical knowledge, pre-service teachers were asked open-ended questions to determine their perceptions regarding the teaching of enterprise education, such as effective teaching strategies for skills development, techniques they will adopt in the future, and specific strategies for embedding enterprise education in subject areas. In the third category, activation, pre-service teachers were asked open-ended questions to determine their perceptions about their potential use of enterprise skills in the future. Questions focused on their likelihood to partake in entrepreneurial initiatives in their schools, to work with another enterprise or social enterprise, or to embed enterprise education in their teaching practice. The final version of the questions used in semi-structured interviews is given in Appendix 1.

### 3.5 Procedure

Pre-service teachers attended the modules over one semester as part of their overall study. A total of eight Irish pre-service teachers who were taking part in the Further Education and Training strand of the degree were invited for interview, of these, seven interviews were completed. A total of 13 Turkish pre-service teachers who were taking part in entrepreneurship in science education were invited for interview, of these, 11 interviews were completed. The interviews were conducted with audio recording, with the permission of the participants. Before the interviews, the participants were informed about the purpose of the research and relevant ethical procedures. Participants were provided with a plain language statement, research information sheet, and informed consent documents. Data were not collected from participants who did not agree to participate on this basis.

### 3.6 Data analysis

The data collected from interviews was analysed using the constant comparative method (Glaser and Strauss, 1967 in Maykut and Morehouse 1994:126). The constant comparative method provides the opportunity to reveal similarities and differences between data categories (Åkerlind et al., 2014) and has been a chosen approach by many phenomenographic researchers (Assarroudi and Heydari, 2016; Boda, 2019; Jacobs and Gravett, 1998). The process involved analysing the data for patterns in the words and phrases in participant responses. Responses often contained multiple pieces of data, which were coded and grouped together as initial categories (for example in category one: ‘solution oriented’). As categories emerged, rules of inclusion were developed to ensure consistency in each category (for example: ‘must relate to opportunity or problem solving’). If a piece of data did not meet the rules for inclusion, a new category was created. This process was repeated until clear categories were present. Finally, propositional statements were developed to capture the essence of each category they represented. This analysis process was also carried out by an independent researcher for trustworthiness of the data and their analysis. An independent researcher reviewed the process of coding the texts and categorizing the codes, meaning the codes and categories reached by the researchers were cross-checked by independent coders with experience in analyzing qualitative interviews to help ensure the reliability of the data (Miles and Huberman 1994). Thus, the inter-coder reliability [(the total number of agreement)**/**(the total number of agreement **+** the total number of disagreements)] was determined as 0.87 for the data obtained from Turkish participants and 0.85 for the data obtained from Irish participants. Since these reliability scores are above the 80% threshold, they are considered satisfactory (Miles and Huberman 1994).

In an effort to test the viability and credibility of these categories and the findings within them, the authors drew on Guba's (1978: 56–57) work for testing the robustness of qualitative data. First, data was checked for internal and external plausibility, ensuring consistency within categories and cohesion among separate categories. This involved reviewing completed data to ensure categories were clearly defined (for example: ‘solution oriented’ only contained comments related to ‘opportunity’ and ‘problem solving’) and that connections existed between categories (for example: ‘creativity’ is related to ‘solution oriented’). Second, the analysis was checked to ensure it was inclusive of the data and information that was available for study. This involved reviewing completed analysis to ensure that all available data and themes were identified and used during the study, including less represented categories which may impact on discourse (evidenced by the inclusion of occurrences such as ‘need for success’). Third, data was tested to establish connections to previous work in the field, and its contribution to this enquiry. Researchers reviewed the categories which emerged and identified links to existing literature (for example: ‘experienced-based strategies’), establishing the relationship to previous work in the area. Finally, a detailed record of the analysis, coding, categorising and presentation of data was kept so that the data was reproducible by another competent judge. Each stage of the process was fully documented to demonstrate these steps. Initial sub-themes identified (for example: ‘curiosity’) were documented as such, before being merged into broader categories (for example: ‘creativity’).

## 4 Findings and discussions

Key categories and findings are now presented using extracts from participant interviews, to address the three categories outlined previously (see summary in Table 1). Throughout the following sections ‘n = ’ is used to denote the number of occurrences of a theme in the data. Findings are followed by overall conclusions and recommendations drawn from the inquiry. As stated in the methodology section, data was analysed using the constant comparative method, and as such will now be presented using propositional statements in an effort to portray the overall meaning of the data categories.

**Table 1:** Summary of findings.

Category	Irish pre-service teachers	Turkish pre-service teachers
Understanding of entrepreneurship	Mixed understanding. Viewed as enterprising mindset and business start-up	Mixed understanding. Viewed as enterprising mindset and business start-up
Understanding of what it means to be enterprising	Transferable skills:	Transferable skills:
	Drive	Drive
	Creativity	Creativity
	Solution oriented	Interpersonal skills
	Interpersonal skills	Research
How should enterprise education be delivered?	Experience-based	Experience-based
	Practical approaches	Practical approaches
	Include teamwork	Include teamwork
	Connect with outside world	Connect with outside world
	Lecturer/teacher support and mentorship	Lecturer/teacher support and mentorship
Potential activation as entrepreneurs and intrapreneurs	Some found the idea of starting a venture appealing	Some found the idea of starting a venture appealing
	Interested in intrapreneurship – but only after establishing themselves in their role	Interested in intrapreneurship – eager to make changes right away
Potential activation as enterprise educators	Promoting ambition, motivation, creativity and problem solving	Promoting creativity, innovation, curiosity and higher order thinking

### 4.1 Participants changing understanding of what it means to be enterprising

In this section participants' changing understanding of what it means to be enterprising is discussed. This includes data on what the terms ‘entrepreneurship’ and ‘enterprising’ mean to participants, followed by their perception of the necessary skills and attributes, and the characteristics they can see in themselves.

#### 4.1.1 Generic skills applied to a variety of contexts

Throughout the interviews, both groups of participants made a variety of comments around their understanding of entrepreneurship and what it means to be an enterprising person. Comments from both groups of participants displayed a mixed understanding of entrepreneurship. Some in line with the ‘enterprising mind-set’ espoused by Gibb (2011) and Rae (2007). For example, Irish participants said: ‘P1 – [entrepreneurs] can think in a different way, think outside the box but also implement those ideas into structures that are already there’, ‘P5 – it means creating new things in a variety of contexts’ and ‘P4 – entrepreneurs take risks to make things happen’. Turkish participants said an entrepreneur is: ‘P2 – An individual who is innovative, who can produce new ideas and solutions to the current problems’. Other comments were more in line with Jones et al. (2014) and Jones and Iredale (2010) assertion that entrepreneurship education is concerned with equipping students with the knowledge, attributes and capabilities required to set up a business. For example, Irish participants said: ‘P1 – entrepreneurship in my opinion is […] learning skills to set up your own business’ and ‘P2 – entrepreneurship is about setting up your own business and having the ability to do that’. Turkish participants said: ‘P6 – It means trading, that is selling something. I see entrepreneurship as being able to express oneself and marketing something to people’, and ‘P11 – Entrepreneurship can be something that does not exist, such as starting a new business or doing something. It also means to produce new things and take risks.’

Participants' understanding of being ‘enterprising’ displayed a range of transferable skills (Bridge et al., 2010) which suggest a similar skill-set to entrepreneurs, applied in different contexts. Irish participants spoke of ‘P5 – always seeing opportunities’, ‘P2 – finding a niche in something they are passionate about’. They viewed being enterprising as a ‘P6 – generic life skill’ or ‘P6 – mindset’ which is focused on ‘P1 – seeing solutions instead of problems’. Turkish participants spoke of ‘P8 – being different … having different perspectives’, ‘P5 – having an idea and research spirit’ and interestingly being ‘P3 – capable of using technology tools well’. This last comment marks a point of difference between Irish and Turkish participants as technology was not mentioned by Irish participants.

Key to this section was participants' views of the key attributes, behaviours and skills which they associated with being enterprising. It is in this area where the first significant differences emerged between the Turkish and Irish cohorts. The most prominent category which emerged from comments in both sets of interviews was **‘Drive’** (Turkey n = 17, Irish n = 19). Irish participants spoke about risk taking (n = 4), resilience (n = 4) and confidence (n = 3). Comments from Irish participants included: ‘P2 – able to take certain risks, to make them and to see if they are worth taking in the long run’, and ‘P4 – you need to be able to take risks to get what you want. You have to be willing to go out of your comfort zone, even if it means talking to people you might not associate with’. Turkish participants spoke of risk taking (n = 5), bravery (n = 3), confidence (n = 5), forward looking (n = 2) and need for success (n = 2). Comments from Turkish participants included: ‘P2 – individuals must consider the difficulties that one might encounter, the consequences of taking risks, s/he must be able to take risks’, ‘P4 – An entrepreneur is a person who can take risks’, and ‘P7 – Entrepreneurs are individuals who are successful and have high self-confidence’.

The second most prominent category in both sets of interviews was ‘**Creativity**’ (Turkey n = 9, Ireland n = 12). Irish participants spoke about creativity (n = 4), doing things differently (n = 4) and making connections. Comments from Irish participants included: ‘P7 – having the ability to think differently and try something different’, and ‘P6 – An enterprising teacher would be creative in their pedagogy or what they teach, or creative in making presentations and activities for their students’. Turkish participants spoke about innovative thinking (n = 3), different points of view (n = 3), curiosity (n = 2) and creative thinking (n = 1).

At this point participants' views of the key attributes, behaviours and skills which they associated with being enterprising begin to diverge. The third most prominent category from Irish participants was that enterprising people are ‘**solution oriented**’. These comments included areas such as problem solving (n = 6), solution oriented (n = 2), and opportunity focused (n = 1). Participants said that enterprising people are ‘P1 – interested in working at problems’, and that ‘P1 – if they see something and notice a bit that isn't quite right, they can see a gap in it and fix it’. The third most prominent category from Turkish participants was **‘interpersonal skills**’ (n = 8). Comments included areas such as communicating effectively (n = 3), decision making (n = 2), negotiating (n = 1), social skills (n = 1) and leadership (n = 1).

Irish participants did produce the ‘interpersonal skills’ category (n = 6), however this was the fourth most prominent and included comments such as leadership (n = 3), negotiating (n = 1), ability to learn (n = 1) and interacting with others (n = 1). Turkish participants identified ‘**research**’ as their fourth and final category (n = 6) with comments indicating they view an enterprising person as a researcher (n = 4), someone who analyses data (n = 1) and a logical thinker (n = 1).

Data above suggests that following participation in enterprise education modules, pre-service teachers have a good understanding of entrepreneurship and enterprise. The data shows relative consistency between cohorts' perceptions in this regard. Both Irish and Turkish participants identified key attributes, behaviours and skills outlined earlier (Rae, 2007; Burduş, 2010; Kirby, 2007; Seelig, 2012; Caird, 1991; Gibb, 2007; Hegarty and Jones, 2008; Arruti and Paños-Castro, 2020) such as: drive, creativity and interpersonal skills, as essential elements of being enterprising, while using slightly different language for each. In one sense, these similarities are not surprising, with Arruit and Panos-Castro (2020) finding pre-service teachers frequently mentioned traits such as creativity and risk taking. What makes the data valuable is that it suggests pre-service teachers in both contexts can identify key transferable skills associated with being enterprising, articulate their meaning, and understand the benefits of developing these skills for use in a variety of contexts, including their own. The only real differences evident from the data was that Irish participants identified more with ‘solution oriented’ while Turkish participants identified with a more ‘logical’ or ‘research’ focused set of attributes. These differences may be attributed to the scientific focus of Turkish participants' qualifications and the more general field of study being taken by Irish participants.

### 4.2 Participants impressions of the pedagogy surrounding enterprise education

In this section participants' impressions of the pedagogy surrounding enterprise education is discussed. This includes data on the strategies or approaches pre-service teachers feel would effectively equip their students with enterprising or entrepreneurial characteristics and their thoughts on the integration of enterprise education into different subject disciplines.

#### 4.2.1 Experience and practice are most important

Following on from their participation in their respective modules, participants were asked to reflect on the strategies which could be employed which they feel would improve the process of enterprise education generally, and to comment on the integration of enterprise education into different subject areas such as mathematics, science, music, etc. Both sets of participants suggested experience-based and practical strategies espoused by authors such as Hindle (2007). Irish participants said ‘P5 – make it practical … simulate things … make it authentic’, ‘P6 – teach students problem solving through case studies and working through real problems’. For example, Participant two said ‘P2 – coming up with a social enterprise’ might help ‘bring it closer and give students an opportunity to experience it [entrepreneurship] for themselves’. Similarly, Turkish participants spoke of arousing the ‘P3 – curiosity of the students’ by ‘P3 – doing research’ on real world tasks. Another participant spoke of the need to increase project work saying: ‘P6 – we must do more projects … making projects and presenting them should be increased’. For example, participants spoke of incorporating ‘P8 – short excursions’ where learners can ‘prepare different materials and discover different things’. Both sets of participants also mentioned the need to incorporate teamwork, with an Irish participant saying it is important to ‘P2 – get people to work in groups … where everyone has a chance to be a leader’. Similarly, a Turkish participant said ‘P9 – teamwork should be encouraged’ so that students can ‘P9 – find a way to accomplish tasks’, ‘P9 – giving them a sense of responsibility. Both sets of participants also highlighted a desire to take their learning outside the traditional class or module boundaries and connect with students from across the wider university and other subject areas. This idea of connecting learning to the outside world was summed up by one Irish Participant who said that lecturers should ‘P5 – encourage students to work with other people outside their class’ so that they ‘P5 – share their ideas and get different viewpoints’. Similarly, one Turkish Participant summed this up saying ‘P6 – there should be inter-school or inter-class activities in order to increase collaboration and competition among students’. Both sets of participants also placed significant value on the role of the teacher/lecturer in the process, although they did frame this in slightly different ways. Irish Participants spoke about the importance of the lecturer being a mentor. They said that the lecturer's experience with project-based and experience-based learning was essential in helping students to ‘P1 – overcome challenges’ and ‘stay focused on their goals’. In this way, they viewed part of the lecturers role as one of support and guidance through the enterprise education process. Turkish participants spoke of the importance of the lecturer being a role-model where they display ‘P4 – enterprising qualities and express them themselves’, while ‘encouraging students’ to develop their own enterprising attributes.

Following participation in the modules, both cohorts of pre-service teachers appear to recognise the value in experiential pedagogies that incorporate real-world tasks, to develop competencies in this area (Henry and Treanor, 2012)**.** This was especially relevant for participants when married with collaborative approaches (Milian and Gurrisi, 2017) which encourage students to work together in teams and share the sense of responsibility. Also evident is the importance of enterprise educator competence (Garnett, 2013), with comments indicating that the ability of the educator to mentor students and emulate enterprising characteristics is an important factor (Otache 2019; Toding and Venesaar 2018). The understanding of the value in experienced-based approaches has important implications for pre-service teachers' future practice as problem-solving and project-based learning have been shown to be more effective than theory-driven approaches (Lackéus, 2015). Pre-service teachers' recognition of the need for role models and mentors is also important as it suggests they understand the value of their role as educators of the future in this regard, offering support and guidance to their students.

### 4.3 Participants potential ‘activation’ as entrepreneurs and/or enterprise educators

In this section participants' potential for activation in the area is explored. This includes data on their likelihood of starting or working with an enterprise, understanding of intrapreneurship, and incorporation of enterprise education.

#### 4.3.1 An entrepreneur? Maybe. An enterprise educator? Yes

Pre-service teachers from both cohorts were asked a series of questions concerning the likelihood of them starting a business, working with another entrepreneur, or acting as an intrapreneur within an organisation. Approximately half (Turkish n = 6, Irish n = 3) of both groups of participants indicated that they would be open to starting their own venture (business or social) within the next 5 years. Many participants in both groups indicated aspirations to be intrapreneurs of some kind in that they would like the opportunity to display their creative capabilities in their future employment and be involved in the development of new ideas. Comments from Irish participants (n = 8) indicated they felt this was something that would take time to feel comfortable doing and they would need time to embed themselves within an organisation first. Comments such as: ‘P2 – if you're only in the door and you start trying to make changes, I don't think it will go very far’; and ‘P4 – for someone new to come in and say, especially someone in a lower position, to say ok we are changing this. It would cause tension and senior members would feel unnerved’, clearly display this need for feeling part of the organisation first. Turksh participants seemed much more confident or assured in their ability to implement change within their future organisations. For example, participant four stated ‘P4 – there are things I want to change related to my own field … would like to design a centre where the students will only be guided by teachers, and where the student will be much more active’. Participant one said ‘P1 – I think that students should be taught life skills’ and that they have ‘P1 – a research-based teaching idea’ to solve this problem.

All Participants across both cohorts indicated a strong interest in incorporating enterprise education in their future teaching. Irish participants' reasons for incorporating enterprise education centred around ambition, motivation, creativity and problem solving. For example, Participant seven sees enterprise education as a way of unlocking students potential, saying ‘P7 – I really think that kids, students, and mature students have so much potential, energy and intelligence to apply their entrepreneurial skills to the world’ and that by ‘P7 – providing them with the right opportunities’, they are given the ‘P7 – chance to use these skills’. Finally, two participants felt they would incorporate enterprise education, but focusing on the creativity and problem solving aspects. Participant three said ‘P3 – I will definitely incorporate it … I would focus on problem solving, or solving problems relevant to students' lives. Another participant said ‘P6 – I would start off small, little things in the classroom around creativity’. Turkish participants were motivated to include enterprise education as a means of improving students' creativity, innovation, curiosity and higher order thinking skills. For example, participant five said ‘P5 – when we incorporate it [enterprise education] into our teaching, students become more curious and as a result understand lessons better’. Participant one said ‘P1 – There is no such thing as everyone will become a teacher or will be an engineer … I should train individuals who are entrepreneurs and who make inventions’. Participant 11 said ‘P11 – I think it should be incorporated. Because science means entrepreneurship. I think if you study science, you should be an entrepreneur, and you should be able to influence people as an entrepreneur. I want to train entrepreneurial individuals in the future’.

This data suggests that following participation in the modules, pre-service teachers from both cohorts are aware of, and may take the opportunity to display their entrepreneurial talents, particularly through intrapreneurship within existing organisations. Interestingly, differences in confidence levels suggest that Turkish pre-service teachers may act on these ideas sooner than Irish pre-service teachers. This may be due to the structured nature of Turkish participants' future role as science teachers when compared with Irish participants' more nuanced role as educators across a range of subjects. Both cohorts were enthusiastic about the prospect of integrating enterprise education in the future, viewing it as a means of drawing out students' motivation, creativity and problem solving skills. Turkish participants in particular were readily able to identify the links between enterprise education and science education (Deveci & Çepni, 2014, 2017; Deveci and Seikkula-Leino, 2018), viewing the incorporation of enterprise education as an opportunity to further develop science teaching as a process of enquiry and discovery.

## 5 Conclusions

The purpose of this study was to examine perceptions of pre-service teachers from Ireland and Turkey towards entrepreneurship and enterprise education, following completion of enterprise education modules on their respective programmes. Data was gathered through participant interviews, around the key areas of content knowledge of enterprise education and entrepreneurship, pedagogical approaches to enterprise education, and entrepreneurship activation and the likelihood of embedding enterprise education in their future teaching. Findings suggest that when exposed to enterprise education in an initial teacher education context and encouraged to experience enterprising skills and behaviours as they relate to their future profession, pre-service teachers from both cohorts can identify a range of transferable skills which are valuable in a multitude of life scenarios, shifting their association of terms such as ‘enterprise’ and ‘entrepreneurship’ away from solely business contexts. While some differences did emerge in the data, there was relative congruence across the Irish and Turkish participants. The practical and embedded nature of their engagement with enterprise education enabled pre-service teachers to see the value in promoting skills such as creativity, drive, leadership, solution orientation, interpersonal skills, and research skills. Participants from both cohorts also strongly supported previous research advocating a focus on experience-based approaches to enterprise education or entrepreneurship education (Bell, 2020; Pittaway et al., 2009; Tiernan, 2016). Pre-service teachers in this study were exposed to many practical elements in their respective programs, however their comments made it clear that participation in more real-world activities involving collaboration and external engagement would further improve the learning experience. Data gathered on participants' future intentions was encouraging. The majority of pre-service teachers indicated that while they may not start their own enterprise in the near future, they would be eager to act as intrapreneurs in their future institutions. Interestingly, Turkish per-service teachers displayed a higher level of confidence in this regard, not feeling the need to establish themselves in their new role before acting as intrapreneurs, like their Irish counterparts. Employers and policy makers see the benefit in developing enterprising mindsets and advocate for the incorporation of enterprise education into all levels of the education system. The data gathered clearly demonstrates the value participants place on the development of enterprising attributes and the importance of including these within education. Both Turkish and Irish participants noted that they will include elements of enterprise education in their future teaching, with a particular focus on social entrepreneurship, empathy, motivation and ambition, creativity and problem solving. As such, participants in this study may benefit from the exposure to enterprise education and be prepared for its potential use in their future teaching. The differences in the perceptions and understanding of enterprise education of the participants from both countries may be attributed to their educational experiences. Turkish participants were being trained as science teachers and have focused on dimensions such as research, curiosity and discovery. Irish participants were being trained as Further Education teachers across a range of subject areas have focused on the more generic aspects of enterprise education. Interesting by way of its absence was the area of employability. Pre-service teachers were concerned with the ability to display their enterprising skills within employment (through intrapreneurship), but less so with the potential benefits to employability gained from participating in enterprise education. This may be due to the perceived stability and permanent nature of teacher employment.

## 6 Limitations

This research has some limitations due to the fact that it is conducted with pre-service teachers studying in different contexts. One of the most serious limitations of the study is that the enterprise education process that the Irish and Turkish pre-service teachers are exposed to includes different variables. However, both cohorts experienced enterprise education based on similar literature and approaches and the study provides important indications of the perceptions of pre-service teachers in two different countries. In addition, it provides insights into pre-service teachers' perceptions as they relate to their current situation and their future practice in each country. Furthermore, this comparative study adds to our understanding of enterprise education programs or modules in international teacher education settings. Additionally, this research is limited to only semi-structured interview data. Participants volunteered for interviews and while participation rates were high it is important to note that the views of those that chose not to participate are not represented in the study. This may have resulted in a minor element of self-selection bias in the findings. In future studies, the enterprise education processes could be examined more comprehensively by using observation, document and quantitative data collection tools. Finally, given that all participants noted that they will include elements of enterprise education in their future teaching, a further longitudinal study investigating how this was achieved, would be of value.

## Declarations

### Author contribution statement

Peter Tiernan and Isa Deveci: Conceived and designed the experiments; Performed the experiments; Analyzed and interpreted the data; Contributed reagents, materials, analysis tools or data; Wrote the paper.

### Funding statement

This research did not receive any specific grant from funding agencies in the public, commercial, or not-for-profit sectors.

### Data availability statement

Data included in article/supplementary material/referenced in article.

### Declaration of interests statement

The authors declare no conflict of interest.

### Additional information

No additional information is available for this paper.

## Appendix A Supplementary data

Interview questions-Draft last version_V2
