# Supplementary material for: Irish and Turkish pre-service teachers understanding and perceptions of enterprise education
Source: Heliyon. 2021 Jul 19;7(7):e07591. doi: 10.1016/j.heliyon.2021.e07591 (PMC8321927; doi:10.1016/j.heliyon.2021.e07591)
Supplement: Supplementary file 1 — Interview questions-Draft last version_V2 [file mmc1.docx]

**Comparison of entrepreneurial knowledge/attitudes of the Turkish and Irish prospective teachers**

| **Interview Form** | |
| --- | --- |
| Pedagogical knowledge | 1. How could we effectively equip students with entrepreneurial or enterprising characteristics? 2. Which teaching methods do you think you will use for entrepereneurship or enterprise education when you start your career ? 3. Which techniques do you think you will use for entrepreneurship when you start your career? 4. What do you think about integration of entrepreneurship or enterprise educatioin in different disciplines (science, music, math etc.)? |
| Content knowledge | 1. What does “entrepreneurship” mean to you? Please explain… 2. What does it mean to be enterprising? Please explain 3. What are entrepreneurial characteristics/enterprising behaviours ? Please explain… 4. Do you see yourself as an entrepreneur/enterprising person? Please explain why… 5. What characteristics of an entrepreneur do you think you have? |
| Activation | 1. Are you likely to start your own (social) enterprsie in the next 5 years? Please explain… 2. Would you work with another start-up business or social enterprise? Please explain… 3. What are your thoughts on intrapreneurship? i.e. entrepreneurship or enterprising behaviour within existing organisations 4. What are your thoughts on the links between entrepreneurship and active citizenship 5. Will you incorporate entrepreneurship/enterprise education in your teaching in the future? Please explain why… |
